# Susceptibility of *Tetranychus urticae* Koch to an ethanol extract of *Cnidoscolus aconitifolius* leaves under laboratory conditions

**DOI:** 10.1186/s40064-015-1127-z

**Published:** 2015-07-11

**Authors:** S Numa, L Rodríguez, D Rodríguez, E Coy-Barrera

**Affiliations:** Programa de Biología Aplicada, Facultad de Ciencias Básicas y Aplicadas, Univesidad Militar Nueva Granada, km 2 Cajica-Zipaquira route, Nueva Granada Campus, Cajicá, Cundinamarca Colombia; Departamento de Química, Facultad de Ciencias Básicas y Aplicadas, Universidad Militar Nueva Granada, km 2 Cajica-Zipaquira route, Nueva Granada Campus, Cajicá, Cundinamarca Colombia

**Keywords:** Acaricide, Fertility, Mortality, LC_50_, Secondary metabolites

## Abstract

One of the main pests of commercial rose crops in Colombia is the phytophagous mite *Tetranychus urticae* Koch. To manage this pest, synthetic chemicals have traditionally been used, some of which are well known to be potentially toxic to the environment and humans. Therefore, alternative strategies for pest management in greenhouse crops have been developed in recent years, including biological control with natural enemies such as parasitoids, predators and entomopathogenic microorganisms as well as chemical control using plant extracts. Such extracts have shown toxicity to insects, which has positioned them as a common alternative in programs of integrated pest management. The objective of this study was to evaluate the effect of an unfractionated ethanolic extract of *Cnidoscolus aconitifolius* leaves on adult females of *T. urticae* under laboratory conditions. The extract was chemically characterized by recording its metabolic profile via liquid chromatography coupled to mass spectrometry, along with tentative metabolite identification. The immersion technique and direct application to rose leaves were used to evaluate the effects of seven doses (10–2,000 µg/mL) of the ethanol extract of *C. aconitifolius* leaves on *T. urticae* females under laboratory conditions. The mortality and oviposition of individuals were recorded at 24, 48 and 72 h. It was found that the *C. aconitifolius* leaf extract reduced fertility and increased mortality in a dose-dependent manner. The main metabolites identified included flavonoid- and sesquiterpene-type compounds, in addition to chromone- and xanthone-type compounds as minor constituents with potential acaricidal effects.

## Background

One of the main pests affecting the rose crops in greenhouses is the phytophagous mite *Tetranychus urticae* (Nachman and Zemek 2002). The spider mites are mainly found on the central and secondary veins of the leaves, and when populations begin to increase, they distribute across the whole leaf (Aponte and McMurtry 1997; Hilarión et al. 2008), reaching high infestation levels and eventually causing leaf drop. This phytophagous species thrives under conditions of high temperature and low relative humidity, displaying explosive growth. It disperses by wind and is also carried on clothing or equipment used in crop management (Hussey and Scopes 1985). Under greenhouse conditions, these pests are distributed in foci due to a reduced ability to move autonomously over long distances (Murillo 1996). It has been noted that certain pesticides can stimulate reproduction, and the use of biological control for predatory mites may therefore be a promising strategy in this type of crop (Powell and Lindquist 1994). The control of this pest has focused on the use of synthetic chemical miticides. However, it is increasingly necessary to supplement this practice with more sustainable tools, for the sake of both the natural environment and humans (Gómez et al. 2004), accompanied by a plan for integrated management of pests and diseases that includes the use of natural enemies, such as predatory mites (usually the family Phytoseiidae), (Osborne et al. 1985; Moraes 1991; Pedigo 1996), and the use of biologically synthesized substances, such as extracts of plant and fungal origin.

Plant extracts, commonly referred to as botanical insecticides, show insecticidal and acaricidal properties at different levels regulating pest populations, despite being unconventional alternatives (Venzon et al. 2005). These extracts contain secondary metabolites produced by plants that have the potential to provide protection against phytophagous organisms and pathogens, and it has been found that these plant extracts also show antifungal properties (Alves 1998). Extracts of *Cnidoscolus aconitifolius* have been studied in several parts of the world, revealing lethal, sublethal and repellent effects on insects (Calderón-Montaño et al. 2011; Ferreira and Moore 2011) and phytophagous mites such as *T. urticae* (Yoon et al.^1998^; Soto et al. 2011; Asharaja and Sahayaraj 2013). Thus, as part of our research on control of economically-important pests and to generate new knowledge that can be applied by growers to manage the phytophagous mite *T. urticae*, the aim of this study was to determine the effect of an unfractionated ethanol-soluble extract of *C. aconitifolius* leaves under laboratory conditions, to provide alternative medium-term strategies to boost compliance with requirements regarding “clean products” for export.

## Methods

### Biological material

Collection of *C. aconitifolius* plants (commonly known as *arnica*) was performed in the Municipality of Agua Azul (Casanare, Colombia) in 2012. Subsequently, the collected plants were identified in collaboration with the Colombian National Herbarium of the Institute of Natural Sciences (National University of Colombia). A voucher specimen was deposited under the collection code COL389957. The individuals of *T. urticae* used for the experiments were collected from a rearing system for this phytophagous species maintained under greenhouse conditions (16.9 ± 2°C and RH of 73.0 ± 0.22%) at the Nueva Granada Military University. From these individuals, a new cohort was obtained to assess 2-days adults, which is appropriate for evaluating the effect of the extracts.

### Extraction of plant material

To prepare the ethanol-soluble extract, plant material (leaves) was dried, ground, and subjected to extraction via maceration (for 7 days) using 96% ethanol, with daily solvent removal.

### High-performance liquid chromatography-mass spectrometry analysis (HPLC–MS)

The ethanol extract was analyzed via liquid chromatography using a Shimadzu LCMS 8030 system (Shimadzu Corp., Nakagyo-ku, Kyoto, Japan). Separation of the components of the extract was performed on a C-18 standard Premier column (4.6 × 150 mm, 5 μm) using an LC–MS system consisting of a separation module equipped with a photodiode array detector (DAD), electrospray ionization (ESI) and a detector with a triple quadrupole mass analyzer. The flow rate was 0.7 mL/min, and for the mobile phases, trifluoroacetic acid (TFA) and 0.005% acetonitrile (ACN) were used. The applied concentration was 1.0 µg/mL in absolute ethanol, and 10 µL of this solution was injected into the LC system. The mass spectrometry method described by Timóteo et al. (2014), Proestos et al. (2006) and Fraser et al. (2014) was used, with the following modifications: a scan using positive and negative ionization mode was performed with an acquisition time of 2–33 min, a mass range of 50–800 m/z, a scan speed of 1,667 μ/s, an event time of 0.5 s, nebulizer gas flow of 1.5 L/min, 350°C interface temperature and DL, and 450°C block temperature. The drying gas flow rate was 9 L/s. The analysis was monitored at wavelengths between 270 and 330 nm. Tentative identification of the major and minor metabolites in the experimental extract was performed through the analysis of mass spectra based on the LC–MS data, complemented with the analysis of retention times. These identifications were supported by the database included in the MassBank Project search engine (free distribution).

### Effect of the *Cnidoscolus aconitifolius* leaf extract on *T. urticae* females

To evaluate the effect of the *C. aconitifolius* leaves extract, an experiment was performed under a completely randomized design with seven concentrations of the plant extract (*Arnica* or *Chaya*), an absolute control and a positive control (Table 1), for a total of nine treatments, each one with three replicates. The experiment was replicated three times over time.

**Table 1:** Description of the treatments applied to *T. urticae* females under laboratory conditions

Treatment	Final dilution concentration (µg/mL)
Absolute control (no application)	–
Positive control (commercial distilled water + acaricide Chlorfenapyr with 24% active ingredient)	–
*C. aconitifolius* [10]	10
*C. aconitifolius* [50]	50
*C. aconitifolius* [100]	100
*C. aconitifolius* [600]	600
*C. aconitifolius* [1,200]	1,200
*C. aconitifolius* [1,600]	1,600
*C. aconitifolius* [2,000]	2,000

Assays were performed at the laboratory of Biological Control (19 ± 0.2°C and 60 ± 2%) of the Nueva Granada Campus at the Nueva Granada Military University, located in Cajicá, Cundinamarca, Colombia.

The experimental unit consisted of a Petri dish 6 cm in diameter, within which a bean leaf disc was placed, rounded with swab. Subsequently, the experimental unit was sealed with stretch film. A total of 20 females of *T. urticae* at 2 days of age were placed on the underside of the leaf bean. Once the extracts were applied in each of the experimental units, they were maintained in the Biological Control laboratory (20.5 ± 1°C and 58.6 ± 3% RH) to complete the recording of data.

Application of the ethanol extract of the leaves of *C. aconitifolius* was performed as follows: each bean leaf disk was immersed in a dilution of the *C. aconitifolius* extract for 15 min, followed by exposure to a gentle stream of air to remove excess of moisture, after which this disc was placed in the experimental unit. Finally, direct application of the extract to the *T. urticae* females present in the experimental unit was performed. Direct application was performed on individuals using an airbrush held at a 20 cm height from the experimental unit, calibrated at 96 drops/cm^2^, with a pressure of 20–30 PSI. The mortality and fecundity of *T. urticae* were recorded at 24, 48 and 72 h using a stereoscope. Thus, the mortality of *T. urticae* individuals was confirmed by a smooth movement no greater than the length of insect body after soft contact with a fine haired brush (Ponte Teles et al. 2011).

### Data analysis

Using the obtained data, daily corrected mortality was calculated for each of the three days of the trial, in the three replicates conducted over time, using Abbott’s formula (Abbott 1925):

$$\% {\text{ Corrected mortality }} = \frac{{\left( {\% {\text{ Mortality in the treatment }} - \% {\text{ control mortality}}} \right) \, \times \, 100}}{{100 \, - \, \% {\text{ control mortality}}}}$$

The corrected mortality of the *T. urticae* females recorded at 72 h was transformed using the function $y = ar\sin \sqrt p$, where *p* is the mortality ratio, and “*y*” is the transformed value. To assess the significant differences between treatments, analysis of variance (ANOVA) and Tukey’s multiple comparison test were used. Logistic regression models were fitted using the generalized linear model technique assuming a binomial distribution and using the logit link function to determine the median lethal dose (LC_50_) at different times of evaluation. The analyses were performed in the statistical language R, version 3.1.2 (Grainge and Ahmesds 1988).

## Results

### Effect of the ethanol extract of the leaves of *C. aconitifolius* on *T. urticae* females

It was found that doses of 600, 1,200 and 2,000 µg/mL of the ethanol extract of *C. aconitifolius* leaves resulted in higher mortality of *T. urticae* females. Although significant differences between treatments (p = 2.2 × 10^−16^) were obtained, the only concentration that did not show a significant difference from the positive control (commercial acaricide) was the highest dose (2,000 µg/mL) (Table 2). This result could indicate that this concentration might be the most effective for producing a formulation for later use in commercial crops to control adult *T. urticae*.

**Table 2:** Percentage of corrected mortality among *T. urticae* females exposed to the ethanol extract of *C. aconitifolius* leaves under laboratory conditions (mean ± standard error) (19 ± 0.2°C and 60 ± 2%)

Treatment	Dose (µg/mL)	Corrected mortality*^,b^
Positive control^a^	–	92.50 ± 1.71 a
*C. aconitifolius* [10]	10	2.50 ± 1.12 e
*C. aconitifolius* [50]	50	5.00 ± 1.29 e
*C. aconitifolius* [100]	100	25.00 ± 1.83 d
*C. aconitifolius* [600]	600	48.33 ± 7.38 c
*C. aconitifolius* [1,200]	1,200	61.67 ± 7.49 bc
*C. aconitifolius* [1,600]	1,600	77.50 ± 1.12 ab
*C. aconitifolius* [2,000]	2,000	92.50 ± 1.12 a

* Values obtaided after 72 h of exposure.

^a^Distilled water + commercial acaricide (active ingredient Chlorfenapyr 24%). Applied dose: 0.4 cm^3^/mL.

^b^Treatments followed by the same letter showed no statistically significant difference according to the Tukey test.

On the other hand, it can be observed in Table 2 that the mortalities induced by doses between 1,200 and 2,000 µg/mL were between 62 and 92%, with the highest concentration (2,000 µg/mL) causing the highest mortality among females of *T. urticae*.

In Table 3, it can be observed that the median lethal dose (LC_50_) decreased with an increasing time of exposure of *T. urticae* females to the plant extract.

**Table 3:** Values (LC_50_) indicating the activity of the ethanol extract of *C. aconitifolius* leaves in inducing the mortality of *T. urticae* females during three time periods under laboratory conditions (19 ± 0.2°C and 60 ± 2%)

LC_50_ (µg/mL) (confidence interval 95%)		
Hours after application	LC_50_ ± standard error	Lower and upper limit of 95% CI
24	1223.637 ± 47.85	1129.86–1317.42
48	990.37 ± 44.24	903.66–1077.08
72	901.25 ± 41.54	819.84–982.67

*CI* confidence interval.

The obtained values of fecundity (Table 4) showed statistically significant differences between the different concentrations of the extract of *C. aconitifolius* leaves at 24, 48 and 72 h (p = 4.04 × 10^−7^, p = 1.20 × 10^−7^, p = 2.20 × 10^−16^, respectively). At 48 and 72 h, it can be observed that the absolute control (no application or 0 µg/mL) displayed significantly higher fecundity than was observed under all doses of the extract. At 48 h, the effect of all doses was similar, while at 72 h, doses between 50 and 2,000 µg/mL induced greater reductions of fecundity compared with the control and the 10 µg/mL dose.

**Table 4:** Mean fertility of *T.* *urticae* females treated with the ethanol extract of *C. aconitifolius* leaves for three days under laboratory conditions (19 ± 0.2 °C and 60 ± 2%)

Concentration (µg/mL)	Per capita fecundity (eggs/day/female) ± standard error^a^		
	24 h	48 h	72 h
Positive control^b^	1.03 ± 0.02 a	1.14 ± 0.06 a	1.28 ± 0.1 a
0	0.56 ± 0.05 b	0.80 ± 0.11 b	1.05 ± 0.04 b
10	0.37 ± 0.04 bc	0.39 ± 0.04 c	0.54 ± 0.07 c
50	0.22 ± 0.04 cd	0.28 ± 0.05 c	0.34 ± 0.03 d
100	0.35 ± 0.06 bc	0.36 ± 0.06 c	0.32 ± 0.06 d
600	0.09 ± 0.02 d	0.15 ± 0.04 c	0.29 ± 0.04 d
1200	0.16 ± 0.04 cd	0.24 ± 0.04 c	0.24 ± 0.04 d
1600	0.27 ± 0.06 cd	0.23 ± 0.07 c	0.17 ± 0.03 d
2000	0.19 ± 0.04 cd	0.20 ± 0.05 c	0.17 ± 0.02 d

^a^Treatments followed by the same letter are not significantly different at α = 0.05.

^b^Distilled water + commercial acaricide (active ingredient Chlorfenapyr 24%). Applied dose: 0.4 cm^3^/mL.

### HPLC-DAD-ESI-MS analysis on plant extract

**Figure 1:**
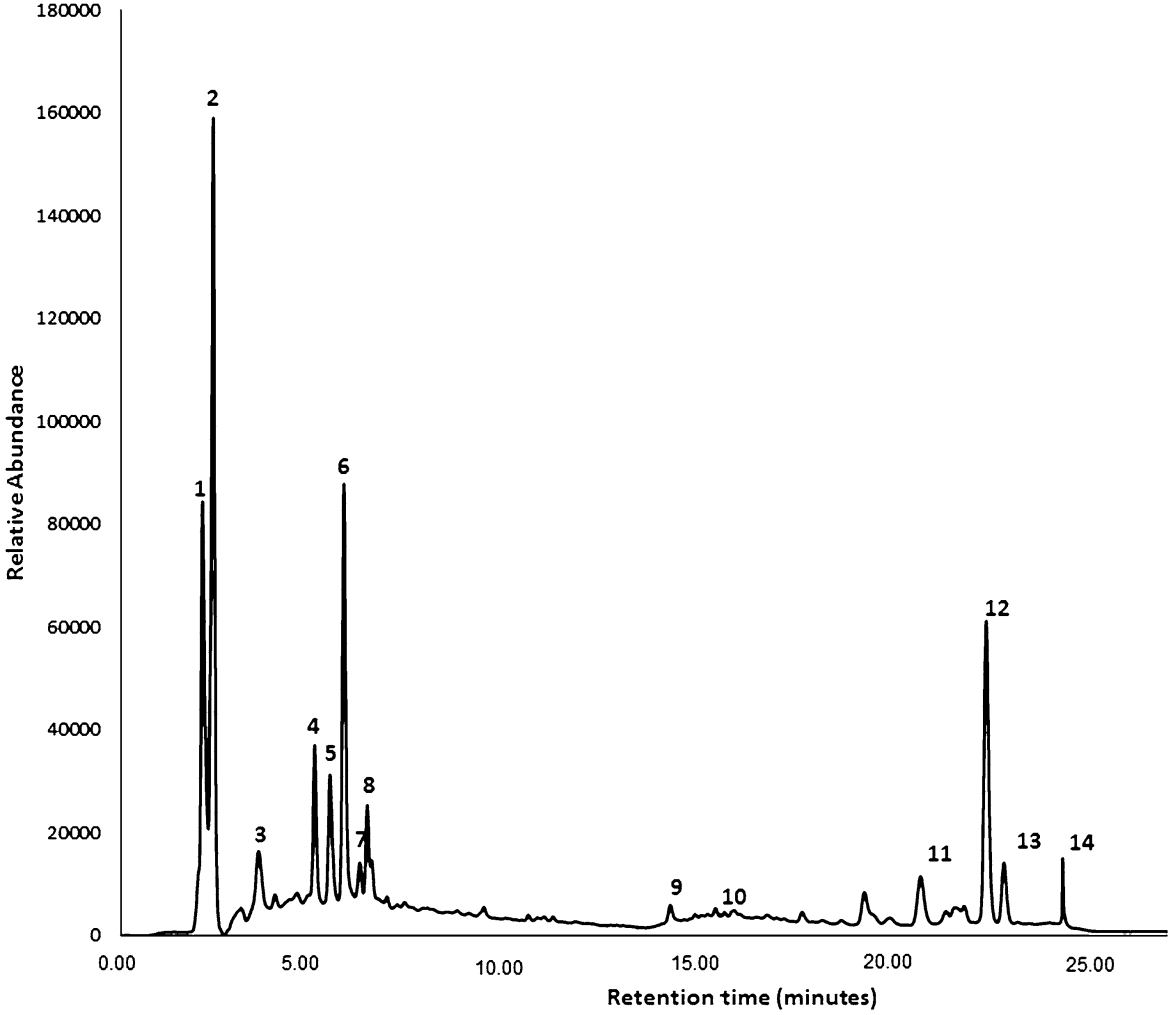
Chromatographic profile obtained via HPLC for the *Cnidoscolus aconitifolius* leaf-derived extract.

The chromatographic profile (Figure 1) of the ethanol extract of *C. aconitifolius* leaves showed two peaks corresponding to the major components **1** and **2**, which were identified as flavonoids (t_R_ = 2.24 and 2.54 min). The third compound identified (t_R_ = 4.89 min) may be a phenolic compound or derivative, which is consistent with the findings reported by Omotoso et al. (2014), who identified phenolic compounds in an *C. aconitifolius* leaf extract using UV–VIS techniques, FTIR and GC–MS.

The detailed analysis of the UV–Vis and mass spectra for each component allowed the tentative identification of 14 compounds in the *C. aconitifolius* leaf extract (Table 5). The main compounds identified were flavonoids (hispidulin sulphate, eucalyptin, and epigallocatechin di-*O*-gallate) and a sesquiterpene (triptofordin D1). The minor compounds identified in the plant extracts were flavonoids ((epi)catechin di-*O*-gallate and acutifolin D), coumarin (fraxetin), a chromone (hamaudol), xanthones (moreollic acid, polyanxanthone C, cadensin G, parvixanthone D) and lignan (tiegusanin F).

**Table 5:** Chemical composition of the ethanol extract of *Cnidoscolus aconitifolius* leaves

No.	Retention time (min)	Name	Compound type	Molecular formula	m/z [M+H]^+^
1	2.24	Hispidulin sulphate	Flavonoid	C_16_H_12_O_9_S	381.03
2	2.54	Eucalyptin	Flavonoid	C_19_H_18_O_5_	327.12
3	4.89	Polyanxanthone c	Xanthone	C_28_H_32_O_4_	433.24
4	5.36	Cadensin g	Xanthone	C_24_H_20_O_10_	469.11
5	5.79	Parvixanthone d	Xanthone	C_24_H_24_O_7_	425.16
6	6.18	(Epi)gallocatechin di-o-gallate	Flavonoid	C_29_H_22_O_15_	611.10
7	6.81	(Epi)catechin di-o-gallate	Flavonoid	C_29_H_22_O_14_	595.11
8	6.91	Fraxetin	Coumarin	C_10_H_8_O_5_	209.04
9	14.63	Acutifolin d	Flavonoid	C_20_H_24_O_5_	345.17
10	15.13	Hamaudol	Chromone	C_15_H_16_O_5_	277.11
11	20.57	Moreollic acid	Xanthone	C_34_H_40_O_9_	593.28
12	22.19	Triptofordin d 1	Sesquiterpene	C_35_H_38_O_11_	635.25
13	23.91	Tiegusanin f	Lignan	C_39_H_38_O_11_	683.25
14	24.45	Unknown	–	–	683.91

## Discussion

According to the results presented in Table 2, the mortality of male *T. urticae* behaved in a dose-dependent manner. This is consistent that the findings reported by Castagnoli et al. (2005), Numa et al. (2011) and Sivira et al. (2011), who observed that increasing the concentration of the extract applied to *T. urticae* adults, the mortality was also increased, in addition to reducing the fertility of the phytophagous females.

A study reported that mortality of *T. urticae* reached a value of 69% using ethanol-soluble *Croton sellowii* extracts, which indicates that there are plants with good acaricide and even repellent effects (Pontes Teles el al. 2011). On comparing the results of the present work, it was found corrected mortalities of 92% (Table 2), which is comparable with that of the positive control used (commercial acaricide with chlorfenapyr as active ingredient).

Table 2 also shows that the mortality caused by *C. aconitifolius* extract is statistically the same to that registered with the commercial acaricide. However, the highest effect of *C. aconitifolius* extract was achieved using the highest dose (2,000 µg/mL). This fact could be rationalized since plant extracts have low concentrations of active components requiring higher concentrations of the extract in comparison to other products.

Additionally, it can also be noted from Table 2 that there were no significant differences in the mortality generated by doses of 10–50 µg/mL, which caused the lowest recorded mortalities. In addition to these results, it is evident from the same table that the mortality induced in *T. urticae* adults did not reach 50% for doses between 10 and 600 µg/mL. This result might indicate that, as established by Gou et al. (1998) and Shi et al. (2008), these concentrations are not appropriate for use as an acaricide for the control of this phytophagous species in commercial crops.

On the other hand, in Table 3, it can be observed that the median lethal dose (LC_50_) decreased with an increasing time of exposure of the *T. urticae* females to the ethanol extract. This result agrees with the findings of Hincapié et al. (2008), Shi et al. (2008) and Teles et al. (2007), who noted that increasing the exposure time of the phytophage to the product can result in toxic interference in biochemical and physiological functions in herbivores, resulting in a decrease of the LC_50_. However, the overlapped confidence interval limits between 48 and 72 h indicated that there were no significant differences.

The results shown in Table 4 indicate that the ethanol extract of the leaves of *C. aconitifolius* causes a reduction in the number of eggs laid per female per day. This is consistent with the results established by Soto et al. (2011), who reported that application of plant extracts may affect the oviposition of *T. urticae* females.

Although the differences between fertility recorded for the different concentrations of the ethanol extract were not significant, a trend towards diminishing fecundity with an increasing dose can be noted in Table 4. These results are consistent with previous reports by Castiglioni et al. (2002), Siviria et al. (2011) and Asharaja and Sahayaraj (2013), who postulated that the number of eggs laid per female decreases when an increasing concentration of a plant extract is applied, which could be associated with possible sublethal effects of extracts on *T. urticae* females. However, it was observed in the tests that eggs laid per alive female were higher in comparison to the evaluated concentrations for *C. aconitifolius* and the positive control (Table 4). This observation is consistent to that reported by Nicastro et al. (2013), who found that a chlorfenapyr resistance was generated by *T. urticae* involving a decreased mortality and an increased fertility. This fact is also consistent to the study by James and Price (2002), who observed that the spray-applied imidacloprid increased the egg production of *T. urticae* by 19–23%.

The two major components observed in the chromatographic profile (Figure 1) corresponded to flavonoids, which agrees with the findings of Awoyinka et al. (2007), Zavoi et al. (2011) and Neha and Jyoti (2013), who indicated that the typical spectrum of flavonoids consists of two peaks with maximum absorption in the 230–340 nm range. The major flavonoids hispidulin sulphate and eucalyptin (Figure 1; Table 5) from plants belonging to the Euphorbiaceae family have been reported to be effective for the control of insects and microorganisms such as bacteria (Calderón-Montaño et al. 2011; Ferreira and Moore 2011; Takahashi et al. 2004) and could have played an important role in the mortalities recorded in Tables 2 and 3. In addition, the presence of terpenes in the extract of the leaves of *C. aconitifolius* (specifically sesquiterpenes, Table 5) indicates that the plant could present an insect repellent effect on *T. urticae*. This result agree with those reported by Yoon et al. (1998), Garcia et al. (1995) and Escalante-Erosa et al. (2004), who found that some sesquiterpenes show a repellent effect against *T. urticae*.

Xanthones have been reported in plants of the Magnoliopsida class, to which *C. aconitifolius* belongs. However, there are no reports of insecticide and acaricide activity for these type of compounds, whereas they have been found to show activity against bacteria, yeasts and crustaceans (Tala et al. 2013). Although the compound hamaudol (chromone-type, Figure 1; Table 5) was a minor constituent of the plant extract, it could be considered as an important metabolite with acaricidal activity. It has been reported by Morimoto et al. (2003) that this compound occurs in plants of the Magnoliopsida class that and it shows antifeedant activity in Lepidoptera. In addition, fraxetin (coumarin, Table 5) could be an interesting metabolite regarding use in the control of *T. urticae*. Moreira et al. (2007) have reported that coumarins show insecticidal activity in several species of Coleoptera.

In conclusion, the unfractionated *C. aconitifolius* leaf-derived extract reduced fertility and increased mortality on *T. urticae* in a dose-dependent manner, possessing a good acaricidal profile to be used for the control of this phytophagous species in commercial crops. The tentative identification of metabolites performed in the present work provides important information supporting the acaricidal activity of the tested plant extract. However, prior to formulation of the ethanol extract, it will be necessary to perform isolation of the compounds it contains (following a bioguided fractionation protocol), in addition to the full chemical characterization and preliminary tests to establish which of the compounds are responsible for the observed acaricidal activity. Additionally, it is recommended that before including this plant extract in an integrated pest management program for *T. urticae*, compatibility tests should be conducted with natural enemies of this phytophagous species, such as predatory mites (*Phytoseiulus persimilis* and *Neoseiulus californicus*). Such tests are necessary because some compounds might also affect predators through behavior modification or lethal and sublethal effects, reducing their ability to predate *T. urticae,* and facilitating the establishment of *T. urticae* in commercial crops where predators are most often released to manage this important pest.
